# A Plant-Derived Alkanol Induces Teliospore Germination in *Sporisorium scitamineum*

**DOI:** 10.3390/jof8020209

**Published:** 2022-02-21

**Authors:** Zongling Liu, Xianruan Lan, Xiufang Li, Haiyun Zhao, Jiaming Gan, Ru Li, Baoshan Chen

**Affiliations:** 1 State Key Laboratory for Conservation and Utilization of Subtropical Agro-Bioresources, College of Life Science and Technology, Guangxi University, Nanning 530004, China; 1808401012@st.gxu.edu.cn (Z.L.); 1608301012@alu.gxu.edu.cn (X.L.); 1908301029@st.gxu.edu.cn (X.L.); 2 Guangxi Key Laboratory of Sugarcane Biology, College of Agriculture, Guangxi University, Nanning 530004, China; 1917304025@st.gxu.edu.cn (H.Z.); 2017402001@st.gxu.edu.cn (J.G.)

**Keywords:** sugarcane smut, teliospore germination, alkanol, transcriptome

## Abstract

Sugarcane smut caused by the basidiomycetes fungus *Sporisorium scitamineum* is a devastating disease for the sugarcane industry worldwide. As the initial step, the smut teliospores germinate on sugarcane buds, and subsequently, the mycelium infects the bud tissues. However, chemical signals that induce spore germination are still unknown. By comparison of the behavior of the teliospores on the buds of both resistant and susceptible varieties, we found that spore germination rates were significantly lower on the buds of resistant cultivars ZZ1, ZZ6, and ZZ9 than on the susceptible varieties GT42 and ROC22. It was found that the levels of hexacosanol and octacosanol were higher on the buds of smut-susceptible varieties than on the smut-resistant varieties. These observations were extended to the smut-resistant and smut-susceptible sub-genetic populations derived from the cross of ROC25 and YZ89-7. In artificial surface assays, we found that hexacosanol and octacosanol promoted smut teliospore germination. Transcriptome analysis of smut teliospores under the induction by octacosanol revealed that genes in the MAPK signaling pathway and fatty acid metabolism were significantly differentially expressed. Overall, our results provide evidence that alkanol plays important roles in smut teliospore germination and thus could be used as a potential marker for smut resistance in sugarcane breeding programs.

## 1. Introduction

Sugarcane is one of the most important crops worldwide for sugar production and a myriad of useful by-products, such as food, fiber, wax, and biofuel [1]. This crop often suffers from sugarcane smut, caused by *Sporisorium scitamineum*, leading to a serious loss of sugarcane yield. Occasionally, teliospores of *S. scitamineum* fall on the surface of sugarcane buds and germinate, then meiosis occurs, leading to haploid sporidia, which, upon forming mating dikaryotic mycelium, can invade sugarcane [2]. A distinct symptom of sugarcane smut is a whip on the top of the stem, which harbors the smut teliospores. Smut teliospores are spread by wind until they eventually land on lateral buds or on the soil, where they survive over winter and become the source of infection in the next year. Chemical control of smut is generally ineffective, while breeding smut-resistant sugarcane varieties has proven to be an efficient and sustainable method of controlling smut [2]. However, breeding smut-resistant varieties is time consuming due to the lack of reliable and efficient genetic markers to screen for smut resistance. Therefore, there is an urgent need to develop a marker for such effective genetic screening.

Smut resistance mechanisms are categorized into structural, biochemical, and physiological resistance mechanisms [2,3,4]. Resistance to sugarcane smut is different in the evaluation of field incidence rates and artificial inoculation [5,6], in which the outer structure of the buds is destroyed, allowing *S. scitamineum* to directly invade the bud interior. This indicates that structural components may play an important role in imparting smut resistance. However, no direct correlation was observed between sugarcane smut resistance and bud morphology, i.e., length, width, shape, and groove, in 15 sugarcane varieties ranging from highly resistant and to highly susceptible [7]. Smut-resistant varieties showed a lower germination rate of smut teliospores on buds than did smut-susceptible varieties [8]. Smut-resistant varieties exhibit more cell wall-associated responses in the early stages of smut infection, such as an increase in lignin, phenolic compounds, cellulose, and arabinoxylan [9]. Enzymes, including peroxidase, ascorbate peroxidase, catalase, superoxide dismutase, β-1,3-glucanase, and malondialdehyde, increase in content in smut-resistant varieties. Furthermore, their levels are correlated with smut resistance, indicating their potential use as markers [5]. Previous studies have reported smut-resistant varieties to exhibit more lignified cells in buds, enhanced accumulation of phenolic compounds, and more trichomes as compared to smut-susceptible varieties [9].

Wax coating on the surface of plants has many functions, such as protection from environmental stress and prevention of water loss [10,11,12]. Wax is an obvious characteristic of sugarcane as well, where it comprises a variable mix of alkanes, alkanols, alkanals, fatty acids, alkenes, aromatic hydrocarbons, ketones, and esters [13,14]. Sugarcane wax has a high application value, such as in medicine, food, cosmetics, and the chemical industry [15]. As a part of the plant, wax plays an important role in biotic stresses [16]. On the one hand, wax provides a firm barrier to prevent pathogen infection; on the other hand, pathogens may stimulate their activity or regulate their growth [17]. For instance, the spore germination rate of *Erysphe pisi* was reported to be 80% at the paraxial end of the blade, while it was 57% at the distal end; this was attributed to the paraxial end of the blade having a higher content of primary alcohols, while the distal end of the blade had a higher content of alkanes and lesser primary alcohols [18]. Wax components of sorghum and maize also influence the behavior and survival of insect pests [19]. The components of dissolved sugarcane wax were determined in an effort to evaluate sugarcane resistance against the sugarcane borer *Eldana saccharina* [12]. It was found that culm surface wax with a high ratio of triacontanol to its corresponding aldehyde was associated with the resistance. Epicuticular wax of sugarcane has been proposed to be a potential genetic marker and predictor of phenotypic traits of economic importance, such as sucrose content, fiber, yield, and susceptibility to pests and diseases, based on data from 122 sugarcane clones. It was shown in this survey that wax and disease resistance were related, although the mechanisms connecting them were still unclear [13].

Given the implication of wax in smut resistance, we investigated the germination behavior of smut teliospores on buds of smut-resistant and smut-susceptible sugarcane varieties or clones that were derived from the same genetic population. Our data showed that higher alkanol content was associated with the higher germination rate of teliospores and smut susceptibility. Comparative transcriptome analysis of the smut teliospore germination process revealed that genes in the MAPK signaling pathway and fatty acid metabolism were involved in response to octacosanol treatment.

## 2. Materials and Methods

### 2.1. Sugarcane Varieties and Sporisorium scitamineum Isolate

Five commercial sugarcane varieties (smut-resistant: ZZ1, ZZ6, ZZ9, and smut-susceptible: ROC22, GT42) and 10 sugarcane clones (smut-resistant: 3–33, 3–69, 23–15, 26–122 and 25–113, and smut-susceptible: 43–51, 45–23, 45–53, 46–33, 79–40) were selected from the F1 population derived from the cross of ROC25 and YZ89–7. Sugarcane varieties and clones were cultivated in the open field with routine management in the Guangxi University sugarcane germplasm nursery (latitude_longitude: 22.5° N 107.7° E, subtropical areas), Guangxi province, China. Smut teliospores were collected from smut-whip obtained in ROC22 in the field of the Guangxi University sugarcane germplasm nursery during July 2021. They were dried at 37 ℃ for 24 h and stored at 4 ℃ before further use. Before smut teliospores were used for inoculation, their germination efficiency was verified with YEPS medium including 1% (*w/v*) yeast extract, 2% (*w/v*) sucrose, 2% (*w/v*) peptone, and 1.5% (*w/v*) agar; germination was observed after incubation at 28 ℃ for 6 h. Smut teliospores with germination rates of >90% were used for subsequent experiments. Buds on the middle third portion of sugarcane stems were photographed with a ruler using a Canon camera. Bud sizes were measured using the Image J (1.8.0) software [20].

### 2.2. Evaluation of the Germination of Smut Teliospores on Sugarcane Buds

The middle healthy stem segments of the five sugarcane varieties and 10 sugarcane clones from the cross of ROC25 and YZ89-7 were selected for this experiment. Stem segments were cut to have single buds in each and soaked in running water for two days. Aliquots of 20 μL of smut teliospore suspension (5 × 10^6^ CFU/mL, 0.01% tween 20) were dropped on the surface of the buds; water (0.01% tween 20) was considered as control. The buds were incubated at 28 ℃ and 90% RH. After 6, 12, 24, and 48 h of inoculation, scales from the outer bud were cut into slices, soaked in lactophenol cotton blue staining solution (10 g carbolic acid, 10 mL lactic acid, 20 mL glycerol, 0.02 g cotton blue, and 10 mL distilled water) for 20 min, and washed with water twice. The slices were examined under a light microscope to count the number of germinations (100 smut teliospores per replicate). Three biological replications were performed.

### 2.3. Scanning Electron Microscopy

Wax on the scales of the outer bud was observed using a scanning electron microscope (SEM, Thermo FEI Quattro S, Waltham, MA, USA). The buds were cut off using a blade, and their outermost scales were separated using tweezers. The scales were fixed in 2.5% glutaraldehyde solution in 0.1 M phosphate buffer (pH 7.2–7.4) at 4 ℃ for 24 h. The samples were then washed with 0.1 M phosphate buffer (pH 7.2–7.4) three times, followed by soaking in an increasing gradient from 10% alcohol to anhydrous ethanol, where soaking with each concentration lasted for 10 min. After dehydration with anhydrous ethanol, ethanol was replaced by tert-butanol. The samples were then frozen to −80 ℃ in a freeze dryer (Labconco FreeZone, Kansas City, MO, USA) for 1 h. The dried samples were treated with spray-gold using a magnetron sputtering instrument (Cressington Sputter Coater 108, Watford, UK) and were observed under the SEM.

### 2.4. Determination of Wax Content

Buds were cut with a blade, and the wax on them was extracted with chloroform for 3 min (ten buds as a biological replication with three procedural replications). Aliquots of 2 μL of tetracosane were added to each sample bud as an internal standard. Samples dissolved in chloroform were air-dried in the fume hood. Aliquots of 40 μL of pyridine (Sigma, P57506, Kawasaki, Japan) and 40 μL of bis (trimethylsilyl) trifluoroacetamide (Solarbio, B8810, Beijing, China) were added into the samples, followed by incubation at 70 ℃ for 40 min for derivatization. This derivatization mixture was dried under a gentle nitrogen stream. Samples were then re-suspended in 1 mL of chloroform, followed by filtration through a 0.45 μm membrane.

Quantitative analysis of wax content was conducted on a GC-MS (Agilent 7890B-7000D, Santa Clara, CA, USA). The capillary column (Agilent 19091S-433UI) was used with helium as carrier gas (1.2 mL/min). The oven was conditioned at 50 °C for 1 min. The temperature was then increased to 170 °C at a rate of 20 °C/min for 2 min, followed by an increase to 300 °C at a rate of 5 °C/min for 15 min. The data thus acquired were analyzed by GC-MS solution software. Peaks were identified with the help of a NIST library and wax standards.

### 2.5. Smut Teliospores Germination on Pure Wax Coating Surfaces

Hexacosanol, octacosanol, palmitic acid, stearic acid, and heptacosane were purchased from TCI (H0342, O0199, P1145, S0163, H0017, Saitama-ken, Japan). Octacosanal was synthesized by octacosanol using pyridinium chlorochromate (Solarbio, P1140) [21]. Briefly, the octacosanol standard (1 mM) was mixed in 50 mL of pyridinium chlorochromate for 1 h at 28 ℃. The mixture was then eluted using silica gel-60 five times. The eluent was dried under a gentle nitrogen stream.

Wax coating on a surface was conducted using the method of Uppalapati [22] with minor modifications. Hexacosanol (C_26_H_54_O), octacosanol (C_28_H_58_O), palmitic acid (C_16_H_32_O_2_), stearic acid (C_18_H_36_O_2_), heptacosane (C_27_H_56_), and octacosanal (C_28_H_56_O) were added to 0.5% polyvinyl formal (TCI, P0614) in chloroform to get solutions with final concentrations of 7 × 10^−3^, 7 × 10^−4^, and 7 × 10^−5^ mol/L (~5, 0.5, and 0.05 μg/cm^2^). Subsequently, 4 mL of each solution was added to 9 mm Petri dishes and was blown dry in the fume hood overnight. Aliquots of 1 mL of teliospores suspension (5 ×10^6^ CFU/mL) were spread onto the surface of the wax coating on Petri dishes. The polyvinyl formal containing no wax purity was considered as control. The Petri dishes were then incubated at 28 °C. Teliospore germination was observed by optical microscope after 6, 12, 24, 48, and 72 h post plating. Each treatment was performed in three biological replications.

### 2.6. RNA Sequencing

Gene expression profiles of germinating smut teliospores were studied using RNA sequencing to elucidate differences in gene expressions under the effect of octacosanol. Smut teliospores that had been treated with octacosanol for 24, 48, and 72 h as described above were washed with sterile water. They were then immediately frozen using liquid nitrogen before being stored at −80℃ for RNA-seq. Samples were named T1, T2, and T3, respectively. Smut teliospores treated with no octacosanol for 48 h were considered as control (named CK). Each treatment was performed in three biological replications. The samples were then sent to Beijing Biomarker Technologies Inc., China for RNA extraction using Trizol reagent, DNase I treatment, cDNA library construction using cDNA-PCR Sequencing Kit (SQK-PCS109), and Nanopore sequencing using Ion Torrent S5 platform.

Raw reads were first filtered, with a minimum average read quality score of 7 and a minimum read length of 500 bp. Clusters of transcripts were obtained after mapping to the reference genome (GenBank: GCA_900002365.1) with mimimap2. Consensus sequences were mapped to the reference genome using minimap2, and redundant transcripts were removed. For gene function annotation, all genes were annotated based on the following databases: NCBI nonredundant protein sequences (NR), Protein Family (Pfam), Swiss-Prot, and Clusters of Orthologous Groups of proteins (COG). Differential expression analysis of two conditions or groups was performed using the DESeq2 R package (1.6.3). Gene ontology (GO) enrichment analysis of the differentially expressed genes (DEGs) was implemented using the GOseq R package. We used KOBAS 3.0 software to evaluate the statistical enrichment of differential gene expression related to the KEGG pathways. Gene set enrichment analysis (GSEA) was performed to analyze the MAPK signaling pathway using GSEA v4.1.0 software [23]. Analysis of trends in gene co-expression was performed using BMKCloud (www.biocloud.net, 15 November 2021).

### 2.7. Quantitative Reverse Transcription PCR Analysis

RT-qPCR was conducted to confirm the gene expression determined by RNAseq. A total of six genes involved in the MAPK signaling and fatty acid degradation pathways were selected for RT-qPCR. The primers were designed using NCBI primer-BLAST tool (https://www.ncbi.nlm.nih.gov/tools/primer-blast/, 10 December 2021). The remaining RNA (DNase I treated) of RNA-seq was used for RT-qPCR analysis. Reverse transcription and qPCR were conducted with PrimeScript™ IV 1st strand cDNA Synthesis Mix (6215A, TaKaRa) and TB Green^®^ Fast qPCR Mix (RR430S, TaKaRa) following manufacturer’s instructions. Inosine 5′-monophosphate dehydrogenase (S10) and SEC65-signal recognition particle subunit (S11) were chosen as internal controls in qPCR analysis [24]. Each treatment was performed in three replications. Primers used in RT-qPCR validation are listed in Table S1. The qPCR data were analyzed using the 2^−^^△△CT^ method [25]. Results from RT-qPCR and RNA-seq were correlated using SPSS 24.0.

## 3. Results

### 3.1. Smut Teliospores Germinates at Varied Rates on the Buds of Smut-Resistant and Smut-Susceptible Sugarcane Varieties

**Figure 1:**
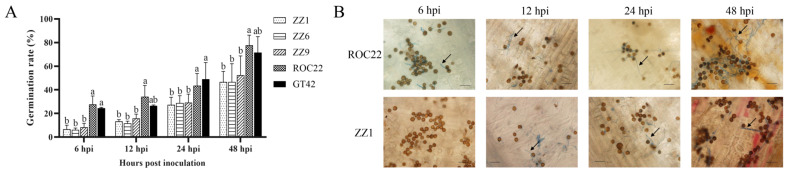
Germination of smut teliospores on buds of different varieties. (**A**) Germination rates on buds of ZZ1, ZZ6, ZZ9, ROC22, and GT42 after inoculation with smut teliospores at 6, 12, 24, and 48 h post inoculation (hpi). (**B**) Observation of germination of smut teliospores on buds 48 h post inoculation. Arrow indicates germ tube emerging from the germinating teliospore; bars = 25 μm. Values followed by the same letter are not significantly different as per Tukey’s test (*p* < 0.05).

Smut teliospores on sugarcane buds were visually observed, and their germination rates were calculated to determine the differences in smut teliospore germination in smut-resistant and smut-susceptible varieties. The percentage of germinated teliospores in smut-susceptible plants (ROC22 and GT42) was higher than in smut-resistant plants (ZZ1, ZZ6, and ZZ9) at 6, 12, 24, and 48 h post inoculation (hpi) (Figure 1A). As shown in Figure 1B, the number of germinated teliospores increased faster in ROC22 than in ZZ1. Furthermore, the fungal hyphae of germinated teliospores were longer in ROC22 than in ZZ1. These observations suggested that the buds of the different varieties differently influenced the germination of smut teliospores.

### 3.2. The Alkanol Contents of the Buds Differ between Smut-Resistant and Smut-Susceptible Sugarcane Varieties

**Figure 2:**
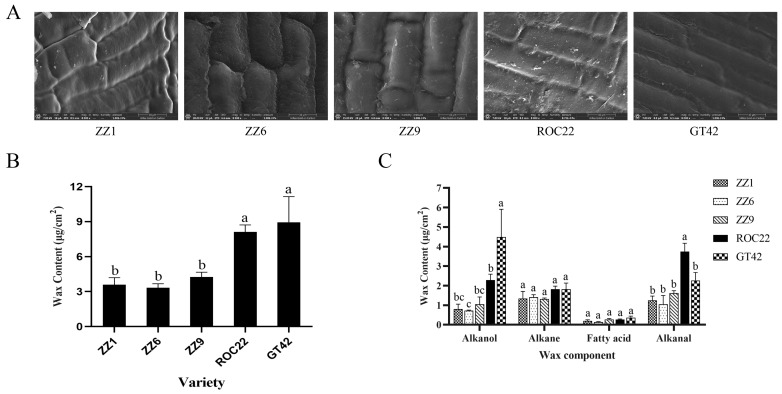
Quantification and observation of wax on buds of different varieties. (**A**) Presence of wax on buds was observed by scanning electron microscopy; bars = 10 μm. (**B**) Total wax content on buds of ZZ1, ZZ6, ZZ9, ROC22, and GT42. (**C**) Content of alkanol, alkane, alkanal, and fatty acids. Values followed by the same letter are not significantly different as per Tukey’s test (*p* < 0.05).

To evaluate the difference in buds of smut-resistant and smut-susceptible sugarcane varieties, bud morphologies, wax content, and wax components were measured. The buds of ZZ1, ZZ6, and ZZ9 were oval, while buds of ROC22 were diamond-shaped; buds of GT42 were pentagonal (Figure S1A). Bud sizes of smut-resistant varieties, ZZ1, ZZ6, and ZZ9, were significantly smaller than those of smut-susceptible varieties, ROC22 and GT42 (Figure S1B). SEM visualization of buds showed that the cellular surfaces in ZZ1, ZZ6, and ZZ9 did not have many wax fragments, while the cellular surfaces in ROC22 and GT42 had a lot of wax fragments (Figure 2A), which indicated more wax on the buds of these varieties. Total wax content in ROC22 and GT42 measured by GC-MS was significantly higher than that in ZZ1, ZZ6, and ZZ9 (Figure 2B), which was consistent with SEM observations.

Components of wax on buds included palmitic acid, stearic acid, pentacosane, heptacosane, nonacosane, tetracosanol, hexacosanol, octacosanol, triacontanol, and octacosanal (Table 1). These components could be divided into four categories: fatty acids, alkanes, alkanols, and alkanals. As shown in Figure 2C, alkanol and alkanal content in ROC22 and GT42 was more than that in ZZ1, ZZ6, and ZZ9. The content of alkanes and fatty acids was not significantly different. These results suggest that alkanol and alkanal may play an important role in smut teliospore germination.

### 3.3. Correlation between Alkanol Content and Teliospore Germination Is Validated in Clones of Sub-Genetic Populations

**Figure 3:**
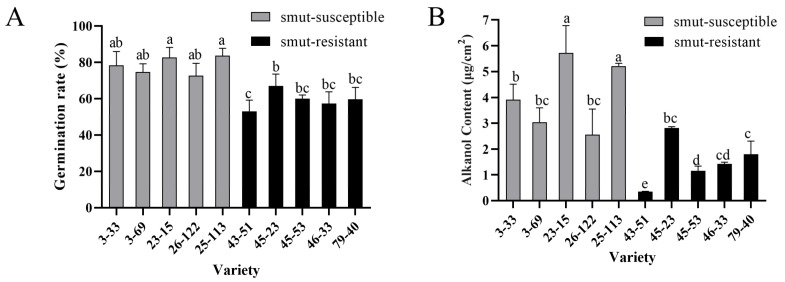
Alkanol content and smut teliospores germination on buds of smut-resistant and smut-susceptible clones of ROC25 × YZ89-7 F1 generation. (**A**) Smut teliospores germination rate on buds of smut-resistant and smut-susceptible clones of ROC25 × YZ89-7 F1 generation. Values followed by the same letter are not significantly different as per Tukey’s test (*p* < 0.05). In this experiment, the concentration of smut teliospore suspension was 5 × 10^6^ CFU/mL and the buds were incubated at 28 °C and 90% RH for 48 h. (**B**) Alkanol content on buds of different clones. Values followed by the same letter are not significantly different as per Tukey’s test (*p* < 0.05).

Field survey of two-year-old perennial ratoons showed distinct smut rates between the smut-resistant clones (0%) and smut-susceptible clones (48.59%–68.08%), although both groups were progenies of the same cross (ROC25 × YZ89-7) (Table 2). Unlike the uniformed small buds for smut-resistant varieties ZZ1, ZZ6 and ZZ9 and big buds for smut-susceptible varieties ROC22 and GT42, the bud morphology of the 10 clones was variable within each group, from small to large, not showing a correlation between the resistance and bud sizes (Figure S1). This bud discrepancy prompted us to assay if there was a difference in influence on teliospore germination and wax content. Indeed, the spore germination rates on the buds of smut-susceptible clones were constantly higher than those on the smut-resistant ones (Figure 3A). Furthermore, the bud alkanol contents of the smut-susceptible clones were also constantly higher than those of the smut-resistant clones (Figure 3B), a result consistent with what was observed in smut-susceptible and smut-resistant varieties.

### 3.4. Hexacosanol and Octacosanol Induce Smut Teliospore Germination In Vitro

**Figure 4:**
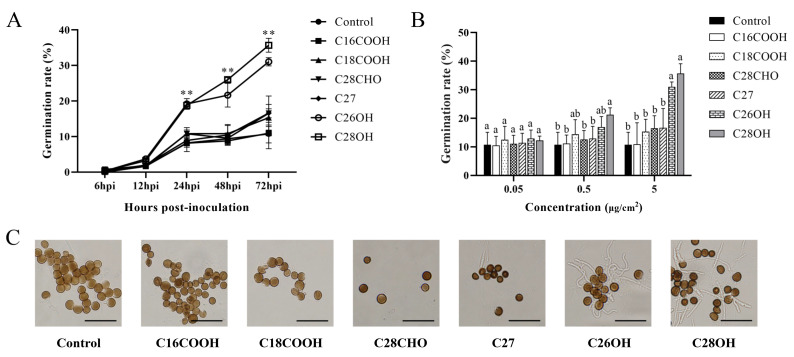
Germination of smut teliospores on pure wax. (**A**) Germination rates of smut teliospores on hexacosanol, octacosanol, palmitic acid, stearic acid, and heptacosane at the concentration of 5 μg/cm^2^. Germination rates of smut teliospores on hexacosanol and octacosanol showed significant difference from the other compounds (**p < 0.01) (**B**) The germination rate of smut teliospores on different concentrations of hexacosanol, octacosanol, palmitic acid, stearic acid, and heptacosane at 72 hpi. (**C**) Observation of germination of smut teliospores on pure wax 72 h after inoculation; bars = 10 μm. Values followed by the same letter are not significantly different by Tukey’s test (*p* < 0.05). In this experiment, the concentration of smut teliospore suspension was 5 × 10^6^ CFU/mL.

We further investigated compounds of the wax in the bud on the germination of smut teliospores. Pure wax components representing fatty acids, alkanes, alkanols, and alkanals were used to treat the teliospores in vitro. In time course experiments from 24 to 72 h, hexacosanol and octacosanol distinguished themselves from the other compounds in positively stimulating the spore germination, while C27 distantly followed, and no effect was observed for palmitic acid, stearic acid, heptacosane, or octacosanal (Figure 4A). In concentration gradients of 5 μg/cm^2^, hexacosanol and octacosanol showed significant difference from the other compounds in positively stimulating the spore germination (Figure 4B).

### 3.5. The MAPK Signaling and Fatty Acid Metabolism Pathways Are Specifically Regulated after Induction by Octacosanol

To further reveal the mechanism by which octacosanol induces germination of smut teliospores, comprehensive gene expression profiles of *S. scitamineum* teliospores at different induction stages (treated with octacosanol after 24, 48, and 72 h, named as T1, T2, and T3, and treated with no octacosanol after 48 h, considered as control, named as CK) were analyzed using high-throughput RNA sequencing. Nanopore sequencing generated 84.29 million clean reads after removal of low-quality reads and those shorter than 500 bp; each sample contained an average of 7.02 million reads (Table S2). On average, 79.18% of reads were considered to be full-length transcripts (Table S2).

**Figure 5:**
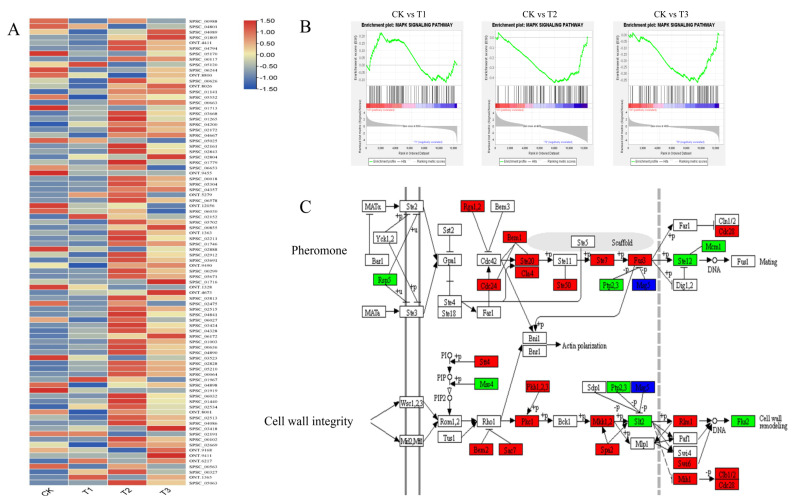
Expression and gene set enrichment analysis (GSEA) of genes in the MAPK signaling pathway. (**A**) Heatmap of gene expression in the MAPK signaling pathway. (**B**) GSEA analysis results for CK vs. T1, CK vs. T2, and CK vs. T3, respectively. The MAPK signaling pathway was significantly upregulated in T2 and T3 (*p* < 0.05). (**C**) Differentially expressed genes of CK vs. T2 in the MAPK signaling pathway. Thirty-three of these DEGs were upregulated, and nine were downregulated. Genes in the red, green, and blue boxes denote upregulated, downregulated, and both up- and downregulated in CK vs. T2, respectively.

Venn analysis (Figure S2) of transcriptomes at different induction stages revealed a large number of differentially expressed genes (DEGs) (Table S3), where the greatest number of DEGs was observed at 48 hpi. The heatmap of gene expression levels showed that genes in the MAPK signaling pathway were upregulated at 48 and 72 hpi (Figure 5A). GSEA analysis showed that the MAPK signaling pathway was significantly upregulated at 48 and 72 hpi (Figure 5B). Of interest was that most of the genes in the pathways involved in pheromone and cell wall integrity were upregulated at 48 hpi (Figure 5C). One of these genes, *SPSC_00663*, encoding *MKK1-MAP* kinase, was strongly upregulated upon induction by octacosanol with log_2_FC values of 4.26, 6.57, and 5.79 at 24, 48, and 72 hpi, respectively. In addition, *SPSC_02892* and *SPSC_04357* were identified to be *RAS2* and *kpp2* genes, and were downregulated and upregulated at 48 and 72 hpi, respectively.

**Figure 6:**
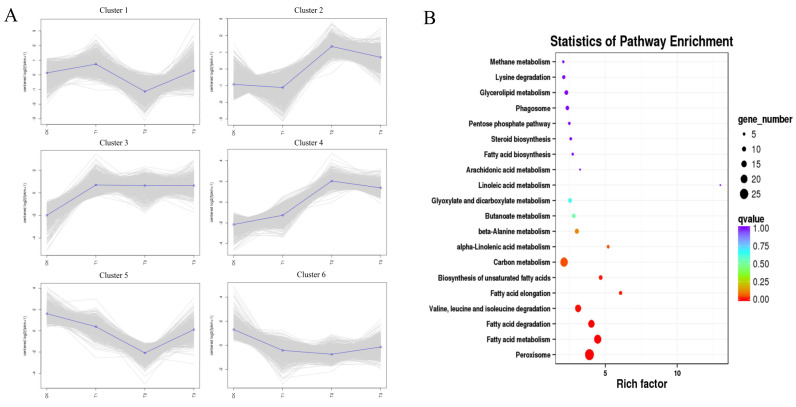
Trend analysis of gene co-expression and KEGG enrichment analysis of cluster 3. (**A**) *k*-means analysis of all gene expression profiles was conducted to obtain six clusters. (**B**) Kyoto encyclopedia of genes and genomes (KEGG) enrichment analysis of cluster 3 showed that pathways of peroxisome; fatty acid metabolism; fatty acid degradation; valine, leucine, and isoleucine degradation; fatty acid elongation, and biosynthesis of unsaturated fatty acids were significantly enriched (*p* < 0.01).

Analysis of the trends in gene co-expression revealed six clusters with distinct pattern based on the gene expression profile (Figure 6A). Genes in the third cluster were continuously upregulated in different treatment stages, and KEGG enrichment analysis revealed the highest number of genes related to peroxisome, fatty acid metabolism, fatty acid degradation, valine, leucine, and isoleucine degradation, fatty acid elongation, and biosynthesis of unsaturated fatty acids (*q* < 0.01), whose rich factors were 3.90, 4.47, 4.03, 3.11, 6.06, and 4.68, respectively (Figure 6B). KEGG enrichment analysis of other clusters revealed no significantly enrichment pathways (*q* > 0.01) (Figure S3). In the fatty acid degradation pathway, *SPSC_01014*, *SPSC_01015*, and *SPSC_01225,* encoding alcohol dehydrogenase (ADH), were highly upregulated, i.e., 2.6- to 7.3-fold under the induction by octacosanol.

**Figure 7:**
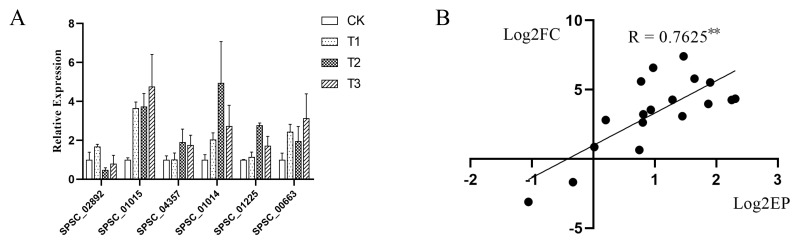
qRT-PCR analysis. (**A**) RT-qPCR analysis of genes in the MAPK signaling pathway (*SPSC_00663*, *SPSC_02892*, and *SPSC_04357*) and three genes encoding alcohol dehydrogenase (*SPSC_01014*, *SPSC_01015*, and *SPSC_01225*). Each treatment was performed in three replications. Inosine 5′-monophosphate dehydrogenase (S10) and SEC65-signal recognition particle subunit (S11) were considered as internal controls. (**B**) Correlation analysis of RNA-seq and RT-qPCR (** *p* < 0.01). The *x*-axis is the logarithm of base two of the relative expression of qRT-PCR, and the *y*-axis is the logarithm of the base two of relative expression of RNA-seq.

Three genes in the MAPK signaling pathway (*SPSC_00663*, *SPSC_02892*, and *SPSC_04357*) and three genes encoding alcohol dehydrogenase (*SPSC_01014*, *SPSC_01015*, and *SPSC_01225*) were selected for RT-qPCR to validate the expression profiles of the transcriptome. As shown in Figure 7, correlation coefficients of RNA-seq and RT-qPCR were 0.7625 (*p* < 0.01). The close relationship implied that sequencing data were reliable.

## 4. Discussion

In previous studies, bud morphology has been implied to contribute to smut resistance in sugarcane [7]. In our study, bud size was initially found to be different between smut-resistant varieties ZZ1, ZZ6, and ZZ9 and smut-susceptible varieties GT42 and ROC22. However, fluctuation in bud size was subsequently observed in both smut-resistant and smut-susceptible clones of the F1 generation of ROC25 × YZ89-7 (Figure S1), implying that bud size alone is not a reliable marker for smut resistance. We then investigated the germination behavior of smut teliospores on the buds of sugarcane and found that the teliospores germinated constantly at a higher rate on the buds of susceptible varieties than on the resistant varieties (Figure 1A and Figure 3A), similar to those observed previously [8,26].

Association of epicuticular wax to disease resistance has been well studied, for instance, seeds of *Zea mays* with higher wax content exhibited higher resistance to *Aspergillus flavus* than did those with lower wax content [27]; leaves of *cassava* with higher wax and greater triterpenoid content similarly exhibited better resistance to *Xanthomonas* [28]. The wax on sugarcane buds was in the form of small scale-like plates and long tubular filaments (Figure 2A), consistent with wax patterns on leaves previously observed using a SEM [29]. It was generally considered that higher wax content would translate into a firmer barrier against pathogen infection. In contrast, we observed that smut-resistant varieties, ZZ1, ZZ6, and ZZ9 had lower total wax content compared to the smut-susceptible varieties, ROC22 and GT42 (Figure 2B), suggesting a possibility that specific wax component may also function to promote smut infection. This assumption was further supported by the fact that susceptible clones also had higher alkanol content compared with resistant clones from the same genetic population (Figure 3B).

Wax is a prominent feature of sugarcane. The components of peel wax measured by GC-MS primarily include alkanes, alkanols, and alkanals, and the major component is octacosanol [13,30]. Wax components on buds determined in this study were similar to those in the peel. In addition, two kinds of fatty acids, palmitic and stearic acids, were also detected (Table 1). We observed that the levels of wax in buds of smut-resistant and susceptible varieties were different. Interestingly, the content of alkanol in wax from smut-susceptible varieties, especially octacosanol, was significantly higher than that in the smut-resistant varieties, which was also observed in sub-genetic populations (Figure 2C and Figure 3B). In agreement with these observations, it has been reported that wax content of sugarcane stalk was associated with resistance, where percentages of octacosanol and hexacosanol were higher in susceptible varieties [13]. These results indicate that alkanol likely plays a key role in the susceptibility of smut.

Our findings provide evidence for the promotion of smut teliospore germination by octacosanol and hexacosanol, while fatty acids, alkanes, and alkanals had no effect on germination (Figure 4A). In other studies, triacontanol promoted the germination of spores of *Phakopsora pachyrhizi* [22] and the development of the conidia of *Blumeria graminis,* especially when supplemented with alkanals, while there were no effects from supplementation with alkanes or alkanols [21]. *Blumeria graminis* has been reported to have reduced appressorium formation due to the absence of alkanal on the leaves of the *glossy11* mutant *Zea mays* [31]. Hexacosanal in the wax of *Hordium vulgare* could induce mycelial formation and appressorium differentiation in *Blumeria graminis* [32]. Overall, these results confirm that wax components influence the germination of spores and different wax components may have varied effects on different kinds of spores.

To investigate the mechanisms of *S.* *scitamineum* response to octacosanol, a transcriptome analysis was performed. Results showed that the MAPK signaling pathway, known to regulate mating, filamentous growth, and pathogenesis in an *S.* *scitamineum* homolog, *Ustilago maydis* [33], was significantly upregulated on being induced by octacosanol (Figure 5B), suggesting its involvement in response to octacosanol and smut teliospore germination. Similarly, genes involved in the MAPK signaling pathway of *U. maydis* have been reported to be involved in response to plant-derived lipids [33,34] and play key roles in pathogenicity as well [35]. The role of the MAPK signaling pathway might be similar in *S.* *scitamineum*, i.e., plant-derived octacosanol, along with other alkanols, could affect pathogenicity by activating the MAPK signaling pathway. In this study, *SPSC_00663* (*MKK1*) and *SPSC_04357* (*kpp2*) were significantly upregulated under octacosanol induction, while *SPSC_02892* (*RAS2*) was downregulated. In *Saccharomyces cerevisiae*, *MKK1* and *MKK2* deletions influence cell lysis [36]. In *U. maydis*, *kpp2* was reported previously to be involved in pheromone and lipid response [33,37] and the *RAS2* gene was observed to be negatively regulated in morphogenesis, pathogenesis, and mating [38]. Moreover, *kpp2* was also required for filamentation in *S. scitamineum* [39]. It indicates these three genes participated in response to octacosanol and smut teliospore germination. Summarily, in smut teliospores, octacosanol and other alkanols may function as signals during the initial infection process in buds.

Trend analysis of gene co-expression showed that genes in the third subgroup, which were continuously upregulated, were significantly enriched in fatty acid-related pathways (Figure 6). Under octacosanol induction, the only carbon source available to smut teliospores was octacosanol, which indicated that genes in the fatty acid-related pathway may be involved in octacosanol utilization. Interestingly, *SPSC_01014*, *SPSC_01015*, and *SPSC_01225*, encoding ADH, which is a primary player in the fatty acid degradation pathway, were significantly upregulated under octacosanol induction. Generally, ADH has the ability to convert ethanol to acetaldehyde in anaerobic respiration; however, it has been reported to exhibit dehydrogenase activities on C10-alkanol [40,41], C16-alkanol [42], and even C30-alkanol [43]. These three genes may thus participate in octacosanol utilization and promote fatty acid-related pathways. Smut teliospores may use alkanols as a carbon source to initiate the infection process on buds.

In conclusion, alkanols in epicuticular wax of buds are a chemical determinant contributing to smut susceptibility by promotion of smut teliospore germination. Of particular interest, octacosanol seems to be the major contributor responsible for induction of teliospore germination, and MAPK signaling pathway and fatty acid metabolism are involved in responding to octacosanol induction. Alkanol content in the buds can be considered as a chemical marker for smut resistance in sugarcane.

## Figures and Tables

**Table 1:** Retention time of wax components.

Standard	Molecular Weight (g/mol)	RT Time (min)
Palmitic Acid (C_16_H_32_O_2_)	256.42	14.916
Stearic Acid (C_18_H_36_O_2_)	284.50	16.418
Pentacosane (C_25_H_32_)	352.70	18.332
Heptacosane (C_27_H_56_)	380.70	19.795
Nonacosane (C_29_H_60_)	408.80	21.079
Tetracosanol (C_24_H_50_O)	354.70	20.286
Hexacosanol (C_26_H_54_O)	382.70	21.493
Octacosanol (C_28_H_58_O)	410.80	23.036
Triacontanol (C_30_H_62_O)	438.80	24.899
Octacosanal (C_28_H_56_O)	408.70	22.164

**Table 2:** Smut field incidence rate of 10 clones of ROC25 × YZ89-7 F1 generation.

Clone	Smut Incidence Rate (%)
3–33	68.08
3–69	58.22
23–15	62.80
26–122	60.60
25–113	48.59
43–51	0
45–23	0
45–53	0
46–33	0
79–40	0

## Data Availability

The raw sequence data reported in this paper have been deposited in the Genome Sequence Archive in National Genomics Data Center, China National Center for Bioinformation/Beijing Institute of Genomics, Chinese Academy of Sciences (GSA: CRA006073 and CRA006106) and are publicly accessible at https://ngdc.cncb.ac.cn/gsa (17 February 2022).

## Supplementary Materials

The following supporting information can be downloaded at https://www.mdpi.com/article/10.3390/jof8020209/s1. Figure S1: Bud areas in all sugarcane varieties used in this study. (A) Bud areas were measured by using the ruler in the Image J software. Values followed by the same letter are not significantly different as per Tukey’s test (*p* < 0.05). (B) A graph of bud size of different varieties. Bud morphology was photographed using Canon camera. Figure S2: Venn diagram of differentially expressed genes in CK vs. T1, CK vs. T2, and CK vs. T3. In CK vs. T1, 365 genes were upregulated, and 139 genes were downregulated; In CK vs. T2, 2704 genes were upregulated, and 1233 genes were downregulated; In CK vs. T3, 650 genes were upregulated, and 226 genes were downregulated. Figure S3: KEGG enrichment analysis of clusters 1, 2, 4, 5, and 6. (A), (B), (C), (D), and (E) represent KEGG enrichment analysis results of clusters 1, 2, 4, 5, and 6, respectively. Table S1: Primers used for RT-qPCR of differentially expressed genes. Table S2: Sample names and clean data statistics from Nanopore sequencing. Table S3: Expression profiles of different expressed genes.
