# Supplementary material for: A Plant-Derived Alkanol Induces Teliospore Germination in Sporisorium scitamineum
Source: J Fungi (Basel). 2022 Feb 21;8(2):209. doi: 10.3390/jof8020209 (PMC8878970; doi:10.3390/jof8020209)
Supplement: Supplementary file 1 [file jof-08-00209-s001.zip › Table S1.pdf]

**Table S1** Primers used for RT-qPCR of differentially expressed genes

| Primer Name | Sense Primer/Anti-sense Primer (5'-3') |
|---|---|
| SPSC_02892 | AACGTGTAGAGAGGTTCCGC/ GTCGCACTTATTCCCGACGA |
| SPSC_04357 | TCACCCTGGAGATTTTGGGC/ GGTTTGCGTTGGGGAACATC |
| SPSC_00663 | TCACGCATACCAGGACGAAG/ CATCTGTGATTGCGGTGTGC |
| SPSC_01014 | CTCATCCTGGCACCCGTC/ ACAGCGTCTTCCGAGTCAAG |
| SPSC_01015 | TCATTGACTCGAAGGAGGCG/ AGTTGATCACCACACGGTCG |
| SPSC_01225 | GTTGGGTCATTTGGGCGTTC/ AGTCGACAAAGACCTCACAAC |
| S10 | CGTTGCAGGACATGGGTGTG/ TTCTCGTAGCTGTGCAGACCA |
| S11 | GAATGCTTGGAGGCATGGGG/ GCGGGTTCATAGGGTCCTTC |
