# Supplementary material for: A Plant-Derived Alkanol Induces Teliospore Germination in Sporisorium scitamineum
Source: J Fungi (Basel). 2022 Feb 21;8(2):209. doi: 10.3390/jof8020209 (PMC8878970; doi:10.3390/jof8020209)
Supplement: Supplementary file 1 [file jof-08-00209-s001.zip › Table S2.pdf]

Table S2 Sample names and clean data statistics from Nanopore sequencing

Sample Name	Read Num	Base Num	N50	Mean Length	Max Length	Full-Length Percentage
CK-1	6705532	5.56E+09	1095	828	146210	84.09%
CK-2	6499795	5.49E+09	1099	844	103886	81.75%
CK-3	7482928	5.99E+09	1029	800	112410	79.23%
T1-1	5257205	8.03E+09	2837	1527	44347	66.80%
T1-2	7081533	8.21E+09	2015	1159	51438	76.12%
T1-3	5808103	7.74E+09	2091	1333	42202	75.85%
T2-1	7741259	8.96E+09	1487	1157	48445	84.25%
T2-2	6800532	8.26E+09	1573	1215	105064	84.41%
T2-3	6854563	7.76E+09	1396	1132	38646	83.47%
T3-1	9256639	6.37E+09	729	688	34692	86.53%
T3-2	5908754	7.87E+09	2117	1331	141404	71.59%
T3-3	8896726	7.23E+09	1130	813	80184	76.07%
