# Supplementary figures and images for: A Plant-Derived Alkanol Induces Teliospore Germination in Sporisorium scitamineum
Source: J Fungi (Basel). 2022 Feb 21;8(2):209. doi: 10.3390/jof8020209 (PMC8878970; doi:10.3390/jof8020209)

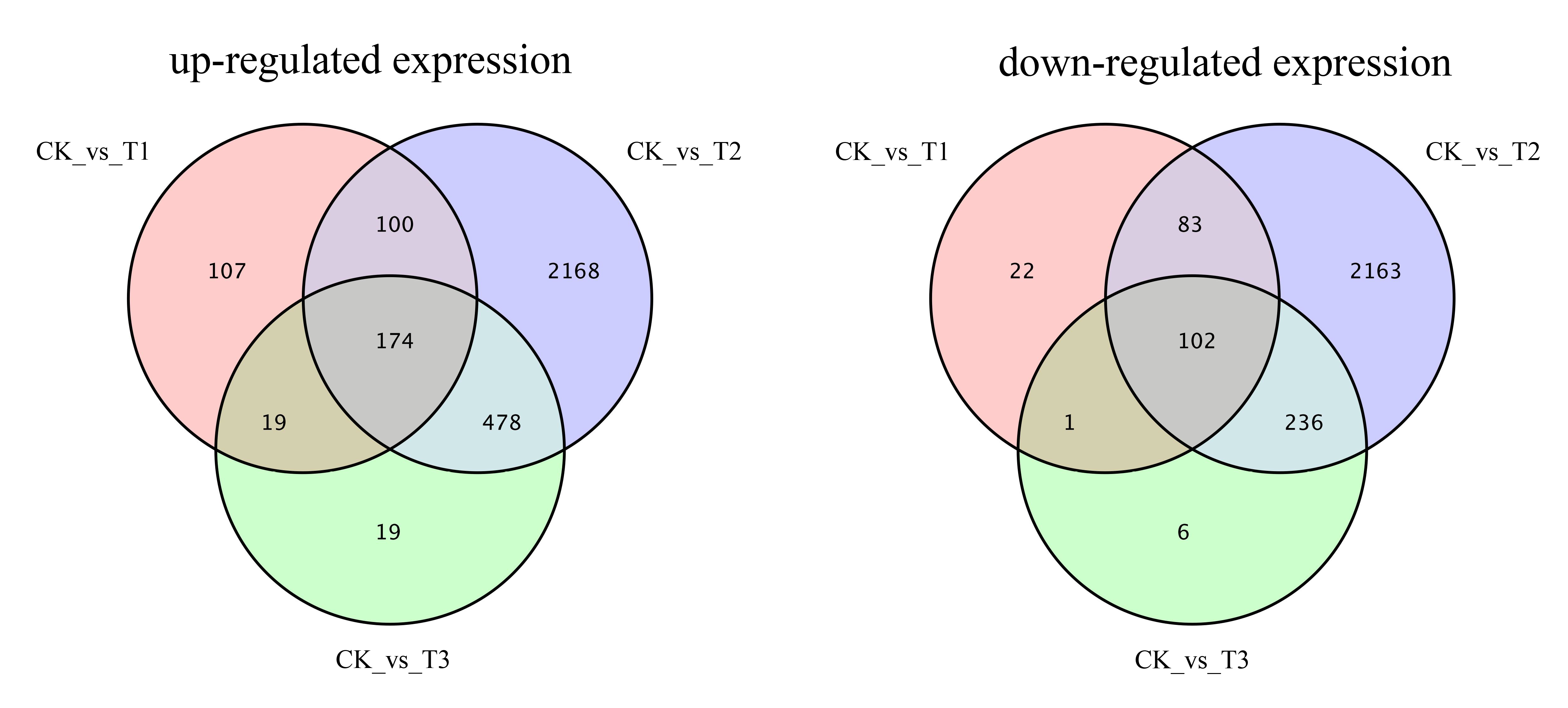

Supplement: Supplementary file 1 [file jof-08-00209-s001.zip › Figure S2.tif]
